# Diversely *N*-substituted benzenesulfonamides dissimilarly bind to human carbonic anhydrases: crystallographic investigations of *N*-nitrosulfonamides

**DOI:** 10.1080/14756366.2023.2178430

**Published:** 2023-02-16

**Authors:** Andrea Angeli, Marta Ferraroni, Alessandro Bonardi, Claudiu T. Supuran, Alessio Nocentini

**Affiliations:** a NEUROFARBA Department, Sezione di Scienze Farmaceutiche, University of Florence, Sesto Fiorentino, Florence, Italy; b Department of Chemistry "Ugo Schiff", University of Florence, Sesto Fiorentino, Florence, Italy

**Keywords:** Carbonic anhydrase, metalloenzymes, *N*-nitrosulfonamides, X-ray crystallography, inhibitor

## Abstract

Carbonic anhydrases (CAs) are a zinc metalloenzymes that catalyse the reversible hydration of carbon dioxide to bicarbonate and proton, pivotal for a wide range of biological processes. CAs are involved in numerous pathologies and thus represent valuable drug targets in the treatments of several diseases such as glaucoma, obesity, tumour, neuropathic pain, cerebral ischaemia, or as antiinfectives. In the last two decades, several efforts have been made to achieve selective CA inhibitors (CAIs) employing different drug design approaches. However, *N*-substitutions on primary sulphonamide groups still remain poorly investigated. Here, we reported for the first time the co-crystallisation of a *N*-nitro sulphonamide derivative with human (h) CA II pointing out the binding site and mode of inhibition of this class of CAIs. The thorough comprehension of the ligand/target interaction might be valuable for a further CAI optimisation for achieving new potent and selective derivatives.

## Introduction

**Figure 1.**
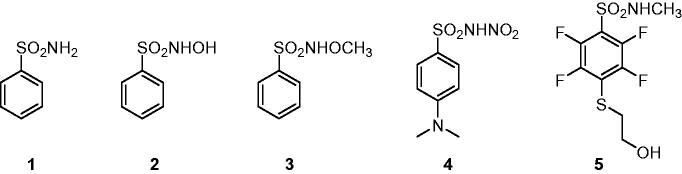
Structures of benzenesulfonamide **1** and *N*-substituted sulphonamides **2**–**5**.

Carbonic anhydrases (CAs, E.C. 4.2.1.1) are a superfamily of metalloenzymes encoded in all life kingdoms by eight genetically unrelated families (i.e. α-, β-, γ-, δ-, ζ-, η-, θ-, and ι-). In human (h), 15 different α-class isoforms are expressed which differ for cellular localisation and distribution in different tissues/organs^1–3^. These enzymes catalysed the reversible hydration of carbon dioxide (CO_2_) to bicarbonate (HCO_3_^–^) and proton that is involved in a multitude of physiologic and pathologic processes^2^^,^^4–6^. In the last decades, the different hCA isoforms have become important therapeutic targets to treat diseases such as glaucoma, oedema, altitude sickness and, more recently, in the treatment of hypoxic cancers^7–9^. In this context, several classes of CA inhibitors (CAIs) have been developed, with the primary sulphonamide compounds, such as benzenesulfonamide **1**, being the most characterised and shown to inhibit the enzyme by directly binding to the zinc(II) ion present in the active site. Nonetheless, these compounds possess many undesired side effects mainly due to their lack of selectivity against the different CA isoforms^10^^,^^11^. For this reason, many efforts have been made for optimising sulphonamide compounds to increase both CA inhibition potency and isoform selectivity, adopting two main drug design strategies: the ring approach consists in varying the aromatic or heteroaromatic scaffolds bearing the sulphonamide group, whereas the tail approach focuses on modifying the one or more “tails” appended to the scaffold bearing the zinc binding moiety for improving the interaction with the outer active site regions of the different isoforms^12^^,^^13^. With the increasing knowledge of the CAI mechanism of action, other classes of selective inhibitors have been developed exploiting different molecular properties, such as coumarin compounds and their bioisosteres, that show a strong selectivity towards the tumour-associated isoforms hCA IX and XII, being though less potent than primary sulphonamide derivatives^14^^,^^15^. A different, less adopted approach aims at substituting one of the hydrogen atom of the primary sulphonamide group to generate different classes of derivatives that exhibit weaker potency than the lead compounds but potentially greater isoform selectivity^16^. The X-ray crystallographic structure of some secondary *N*-substituted sulphonamides (**2** and **3**, Figure 1) complexed with hCA II was reported by Di Fiore et al. demonstrating that, as the primary sulphonamide parent compound, these compounds are still able to bind the zinc ion in the enzyme active site in the deprotonated form^17^. Our group lately discovered the *N*-nitro aromatic sulphonamide as a novel CAI chemotype exhibiting effective as well as selective inhibitory action against hCA IX, the main tumour-associated hCA, among several human isoforms^18^^,^^19^. However, data are not present regarding their binding mode to the target. We report here for the first time the X-ray crystallography of hCA II in adduct with a *N*-nitro benzenesulfonamide derivative, that is the 4-(dimethylamino)-N-nitrobenzenesulfonamide **4** (Figure 1), thus elucidating the mechanism of CA inhibition of this class of compounds.

## Results and discussion

**Scheme 1.**
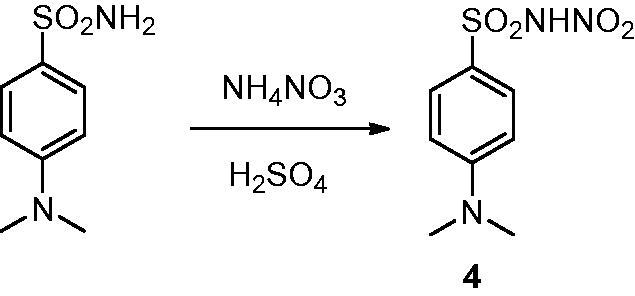
Synthetic route to obtain the *N*-nitrosulfonamide derivative **4**.

The synthetic strategy to obtain the *N*-nitrosulfonamide derivative **4** consists in the chemoselective mononitration of aminosulfonamide according to the route previously reported by our group^18^^,^^19^ and highlighted in Scheme 1.

Notably, compound **4** demonstrated a low micromolar inhibitory efficacy against the tumour-associate isoforms hCA IX and XII, specifically 13-fold greater than against the ubiquitous, commonly off-target, isoforms hCA I and II. On the other hand, the *N*-hydroxy and N-methoxy derivatives **2** and **3** had showed an improved, still low micromolar, action against hCAs I and II, a weaker efficacy against hCA IX, finally inducing hCA XII inhibition similarly to derivative **4** (Table 1).

**Table 1.** Inhibition data of human CA isoforms I, II, IV, IX, and XII with compounds **1**–**4** and **AAZ**^17^^,^^19^^,^^20^.

*K*_I_ (µM)^a^					
Cmp	hCA I	hCA II	hCA IV	hCA IX	hCA XII
**1^b^**	0.086	0.101	7.96	0.097	0.090
**2^c^**	2.73	5.47	24.6	60.3	1.53
**3^c^**	>1000	8.96	39.5	64.3	8.32
**4^d^**	58.3	64.2	11.0	4.5	3.9
**AAZ**	0.25	0.012	0.074	0.026	0.006

^a^ Mean from three different assays, by a stopped flow technique (errors were in the range of ±5–10% of the reported values).

^b^ Inhibition data from Ref.^19^

^c^ Inhibition data from Ref.^16^

^d^ Inhibition data from Ref.^17^

In contrast, the parent benzenesulfonamide **1** (as well as the standard **AAZ**) exhibits a much more potent inhibitory action with inhibition constants (K_I_s) in the low-medium nanomolar range against all CAs reported in Table 1, with the exception of hCA IV. Nonetheless, as stated above compound **1** did not report any relevant isoform selectivity.

Comparing the inhibition profile of the three *N*-substituted derivatives **2**–**4**, it is clear that the group incorporated on the sulphonamide moiety modulate the selectivity against an isoform rather than another. This makes the knowledge of the binding mode of *N*-nitrosulfonamides to the target CAs crucial for a drug optimisation strategy aimed at increasing potency and isoform specificity of this recent class of inhibitors.

**Figure 2.**
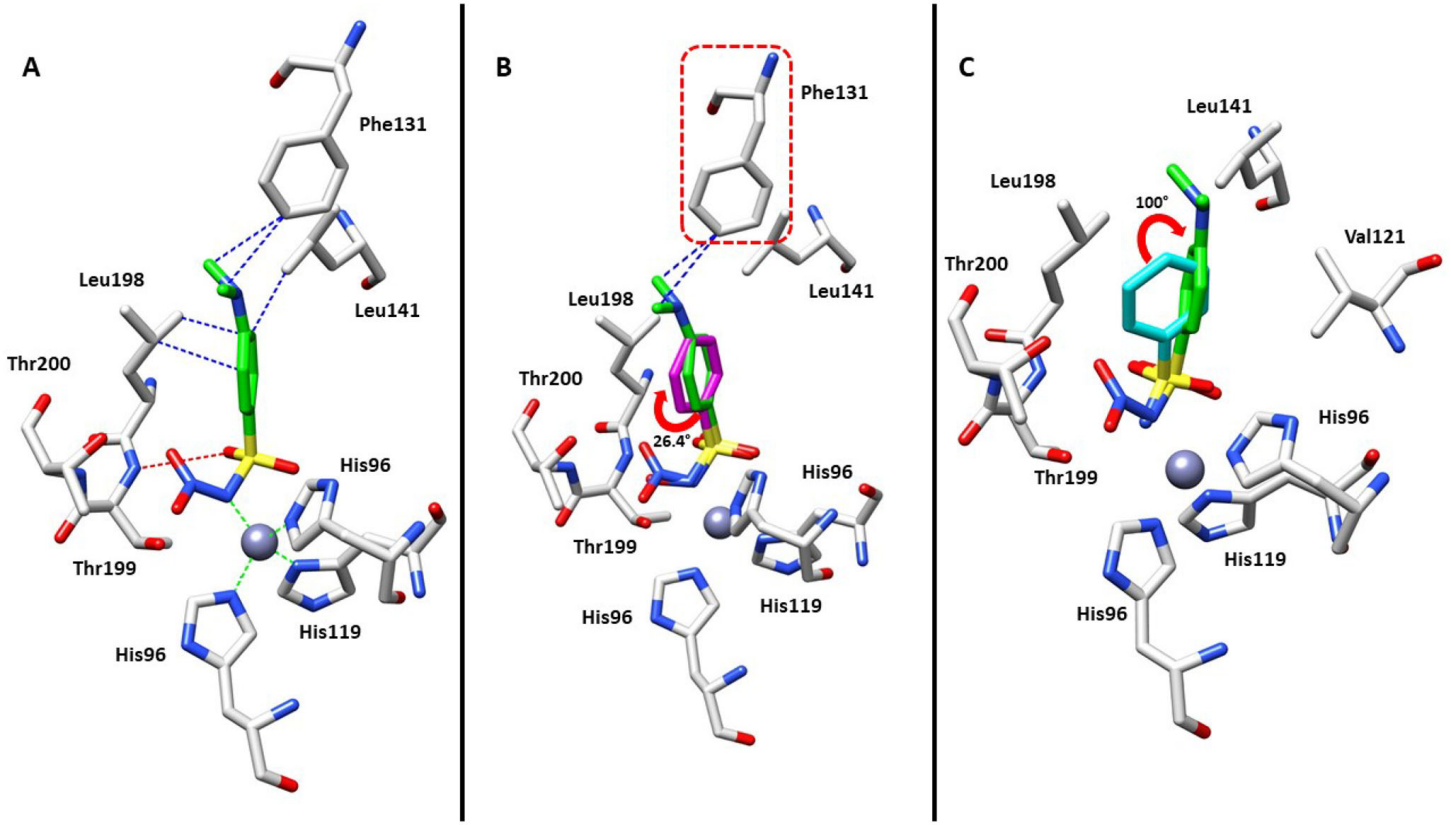
(A) Active site region of the **4**-hCA II complex (PDB: 8BZZ). Hydrogen bonds (red), van der Waals interactions (blue), and zinc interactions (green) are also shown. (B) Structural superposition between **4**-hCA II (green) and **2**-hCA II (magenta, PDB: 3T5U)^17^ bound to the active site of protein. (C) Structural superposition between **4**-hCA II (green) and **1**-hCA II (cyan, PDB: 2WEJ)^21^ bound to the active site of protein.

The high-resolution crystal structure (1.07 Å) of hCA II in complex with the *N*-nitro benzenesulfonamide **4** was attained according to a well-defined ligand electron density, fully compatible with the soaked inhibitor (Figure S1, Supporting Information) bound to the zinc(II) ion. The ligand showed a superimposable binding mode to that previously reported for the *N*-hydroxybenzenesulfonamide **2**^17^, although the –NO_2_ group elicits a greater steric hindrance with respect to the –OH moiety. In detail, the nitrogen atom of sulphonamide group in the deprotonated form, displaces the water molecule present in the native enzyme, and becomes the fourth zinc ligand with a tetrahedral geometry (Figure 2(A)). It should be stressed that this inhibition mechanism resembles that of the parent primary sulphonamides, with other typical interaction with amino acid residues nearby the zinc ion, such as H-bond between a sulphonamide oxygen atom and the backbone NH of Thr199.

**Figure 3.**
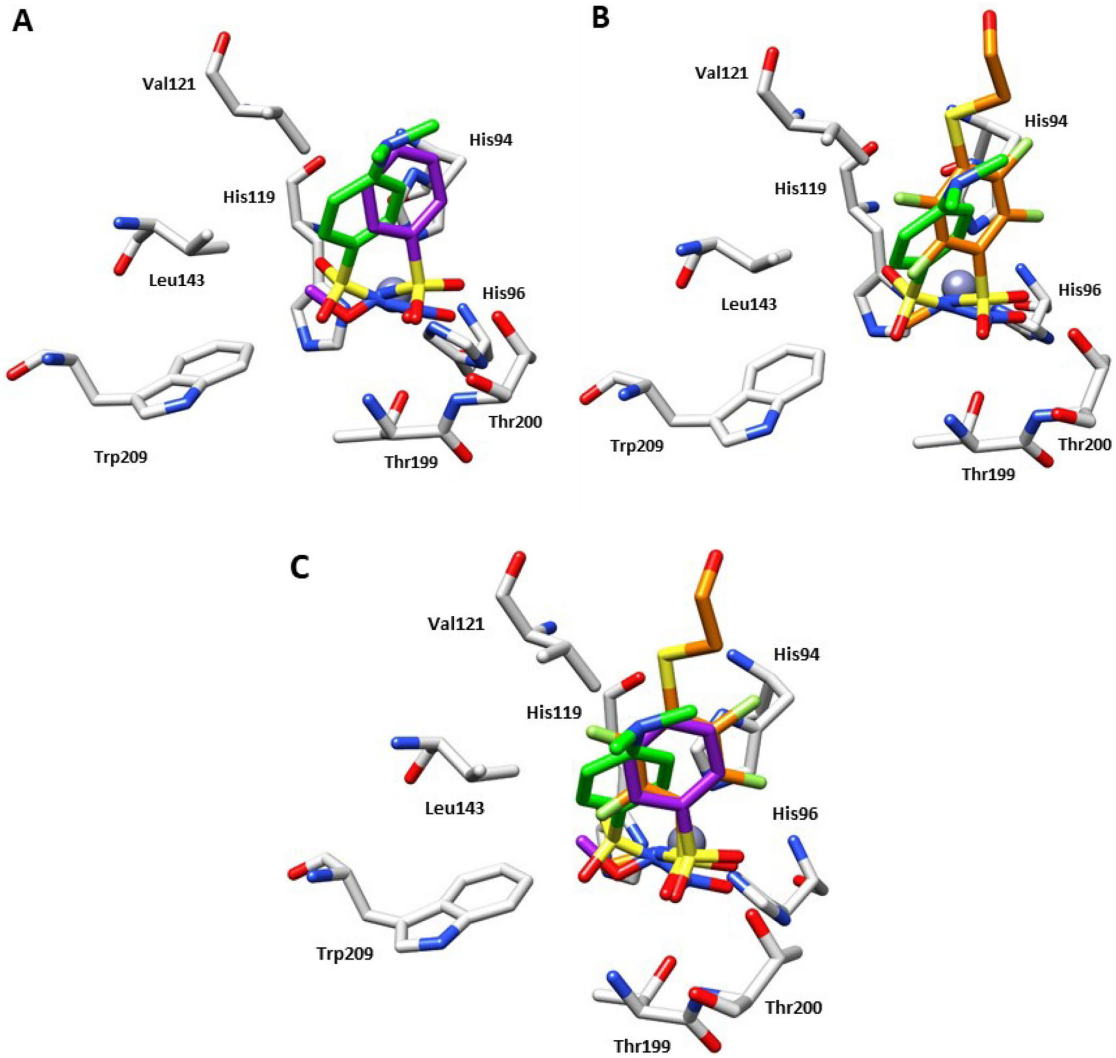
(A) Structural superposition between **4**-hCA II (green) and **3**-hCA II (purple, PDB: 3T5Z)^17^ bound to the active site of protein. (B) Structural superposition between **4**-hCA II (green) and **5**-hCA II (orange, PDB: 7AEQ)^23^ bound to the active site of protein. (C) Structural superposition between **4**-hCA II (green), **3**-hCA II (purple), and **5**-hCA II (orange) bound to the active site of protein.

However, inhibitor **4** was introduced in the model with a partial occupancy of approximately 0.70. The aromatic ring engages several hydrophobic interactions with the side chains of Leu141 and Leu198 and, in addition, the dimethylamino group interacts with Phe131 through van der Waals interactions (Figure 2(A)). The nitro group is located in a hydrophilic pocket formed by the amino acid residues Thr199 and Thr200 (Figure 2(A)). The structural superposition between the adducts of hCA II with compounds **4** and **2** does not show significant differences in the binding mode or in the location inside the active site, except for a 26° rotation of the aromatic ring, probably resulting from a more extended set of van der Waals interactions that the dimethylamino group of compound **4** forms with the side chain of Phe131 (Figure 2(B)). Instead, the superimposition of the hCA II adducts with derivatives **1** and **4** highlights significant differences in the binding mode, mainly existing in the position of the aromatic ring within the active site cavity. In fact, the *N*-substitution induces a steric hindrance obligating the benzene ring to rotate (100° approximately) for compound **4** compared to the lead **1**, resulting in the loss of important hydrophobic interactions with the side chain of Val121 (Figure 2(C)). From a thorough investigation of electrostatic potential surface map around the zinc ion, it is possible to distinguish two different pockets: a hydrophilic pocket formed by the residues Thr199, Thr200, and His96, as mentioned above, and a hydrophobic one defined by Trp209, Val143, and Val121 which is known to represent the binding pocket for the enzyme substrate CO_2_^22^. The different interactions of *N*-substituted sulphonamides with these two pockets might be exploited to modulate potency and selectivity of action. As a matter of fact, relevant information in this context came from comparing the binding mode of compound **4** to that of the *N*-methoxy sulphonamide **3** and *N*-methyl sulphonamide derivative **5** (Figures 1 and 3)^17^^,^^23^. Hydrophobic substituents appended at the sulphonamide moiety, such as in compounds **3** and **5** accommodate in the CO_2_ binding pocket (Figure 3(A,B)), whereas hydrophilic substituents such as in compounds **2** and **4** accommodate in the pocket lined by Thr199, Thr200, and His96, showing a contrariwise orientation of sulphonamide moiety, where, however, the latter still coordinates the zinc ion by a tetrahedral geometry. As a result, significant differences also exist in the orientation of the benzene ring which is completely shifted towards the entrance of the binding cavity (Figure 3(C)).

## Experimental protocols

### Chemistry

The *N*-substituted sulphonamide derivatives **2**–**4** were prepared as reported in the literature^17^^,^^18^.

### Carbonic anhydrase inhibition

The CA inhibition has been assayed by the CO_2_ hydrase method implemented by Khalifah^24^.

### Crystallisation and X-ray data collection

Crystals of hCA II were obtained using the hanging drop vapour diffusion method using 24-well Linbro plate. Two microlitres of 10 mg/ml solution of hCA II in Tris–HCl 20 mM pH 8.0 were mixed with 2 µl of a solution of 1.5 M sodium citrate, 0.1 M Tris pH 8.0 and were equilibrated against the same solution at 296 K. The complexes were prepared by soaking the hCA II native crystals in the mother liquor solution containing the inhibitors at concentration of 10 mM for 15 min. All crystals were flash-frozen at 100 K using a solution obtained by adding 15% (v/v) glycerol to the mother liquor solution as cryoprotectant. Data on crystals of the complexes were collected using synchrotron radiation at the XRD2 beamline at Elettra Synchrotron (Trieste, Italy) with a wavelength of 1.000 Å and a DECTRIS Pilatus 6M detector. Data were integrated and scaled using the program XDS^25^.

### Structure determination

The crystal structure of hCA II (Protein Data Bank (PDB) accession code: 4FIK) without solvent molecules and other heteroatoms was used to obtain initial phases using Refmac5^26^. Five percent of the unique reflections were selected randomly and excluded from the refinement data set for the purpose of Rfree calculations. The initial |Fo – Fc| difference electron density maps unambiguously showed the inhibitor molecules. The inhibitor was introduced in the model with 1.0 occupancy. Refinements proceeded using normal protocols of positional, isotropic atomic displacement parameters alternating with manual building of the models using COOT^27^. The quality of the final models was assessed with COOT and RAMPAGE^28^. Atomic coordinates were deposited in the PDB (accession code: 8BZZ). Graphical representations were generated with Chimera^29^.

## Conclusions

This work enriches the structural information over the binding mode of *N*-substituted sulphonamides to the target hCAs. These results and comparison clearly indicate that N-substitution of primary sulphonamide derivatives as a drug design strategy can compete with the more exploited ring and tail approaches for yielding isoforms selective CA modulators. The knowledge of the CA binding mode of *N*-nitrosulfonamides will be crucial for a drug optimisation process aimed at increasing potency and isoform specificity of this recent class of inhibitors, as potential agents for the treatment of various diseases in which CAs are involved, among which cancer and infections.

## Supplementary Material

Supplemental Material
