# Supplementary material for: Diversely N-substituted benzenesulfonamides dissimilarly bind to human carbonic anhydrases: crystallographic investigations of N-nitrosulfonamides
Source: J Enzyme Inhib Med Chem. 2023 Feb 16;38(1):2178430. doi: 10.1080/14756366.2023.2178430 (PMC9946301; doi:10.1080/14756366.2023.2178430)
Supplement: Supplemental Material [file IENZ_A_2178430_SM2490.pdf]

Supplementary Material for

Diversely N-substituted benzenesulfonamides dissimilarly bind to human carbonic anhydrases: crystallographic investigations of N-nitrosulfonamides

Andrea Angeli,[a]* Marta Ferraroni,[b]* Alessandro Bonardi,[a] Claudiu T. Supuran,[a] Alessio Nocentini[a]

[a] NEUROFARBA Department, Sezione di Scienze Farmaceutiche, University of Florence, Via Ugo Schiff 6, 50019 Sesto Fiorentino, Florence, Italy.
[b] Department of Chemistry "Ugo Schiff", University of Florence, Via Della Lastruccia 3-13, I-50019, Sesto Fiorentino, Italy.

Index

Summary of Data Collection and Atomic Model Refinement Statistics for hCAII *S2*
Figure S1 *S3*

2. Summary of Data Collection and Atomic Model Refinement Statistics for hCAII

	**hCAII + 4**
PDB ID	8BZZ
Wavelength (Å)	1.000
Space Group	P21
Unit cell (a, b, c, α, β, γ) (Å,°)	42.38, 41.50, 72.16<br>90.00, 104.39, 90.00
Limiting resolution (Å)	69.89-1.07 (1.07-1.10)
Unique reflections	106121 (7516)
Rmerge (%)	4.8 (59.1)
Rmeas (%)	5.3 (66.7)
Redundancy	5.47 (4.25)
Completeness overall (%)	98.6 (94.4)
<I/σ(I)>	17.05 (2.46)
CC (1/2)	99.9 (76.9)
**Refinement statistics**	
Resolution range (Å)	69.89-1.07
Rfactor (%)	12.28
Rfree(%)	14.68
r.m.s.d. bonds(Å)	0.0214
r.m.s.d. angles (°)	2.2618
**Ramachandran statistics** (%)	
Most favored	96.1
additionally allowed	3.9
outlier regions	0.0
**Average B factor (Å^2)**	
All atoms	14.561
inhibitor	15.883
solvent	26.240

3. Figure S1:

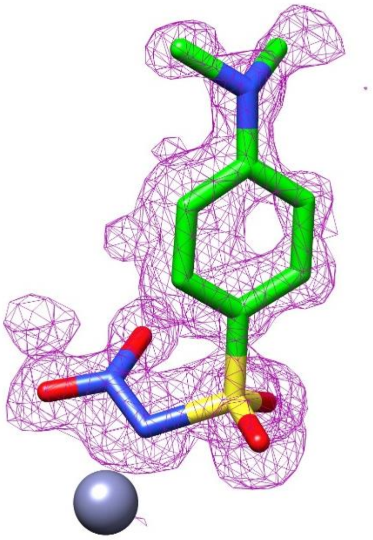

Electron density of inhibitor **1** bound to zinc (grey) in hCA II active site. $2F_o$-F_c maps and contoured to the 1.0 σ level.
